# Medical Students as an Elective Trainee: An Experience

**DOI:** 10.31729/jnma.8223

**Published:** 2023-07-30

**Authors:** Shree Krishna Poudel, Nishan Bhandari, Bindira Adhikari

**Affiliations:** 1 Nepalese Army Institute of Health Sciences, Sanobharyang, Kathmandu, Nepal; 2 Chitwan Medical College and Teaching Hospital, Bharatpur, Chitwan, Nepal

**Keywords:** *career choice*, *communication*, *leadership*, *medical students*

## Abstract

Elective courses beyond the curriculum in medicine refer to additional educational opportunities or experiences that medical students can pursue outside of their required coursework. In the 21st century, with a highly competitive and evolving educational landscape, the importance of elective courses has grown significantly. Completing elective courses can provide several advantages that help individuals stand out and differentiate themselves from their peers. The importance of elective courses for medical students helps to become more competent and self-reliant, improve communication and interpersonal skills and develop leadership qualities. An elective course for a month helped us understand three sectors: personality development, career orientation, and research and publication. This article focuses on the importance of early exposure to careers in research and academia for going beyond the scope of the average medical student.

## INTRODUCTION

An elective course is a course that students can choose to take based on their personal interests or academic goals in a small-group learning setting.^1^ Adherence to academic textbooks and the atmosphere of hospitals limits the medical students from engaging in activities beyond the medical curriculum. A common apprehension exists that involvement in extracurricular pursuits could hinder their educational advancement and future professional prospects.^2^ However, it is crucial to consider the importance of leadership skills in imparting quality health service alongside an understanding of medical concepts.^3^ A previous study mentioned following characteristics defining a good doctor; general interpersonal qualities, communication and patient involvement, medical competence, ethics, medical management, teaching, research, and continuous education.^4^ These skills are important factors to ensure the provision of high-quality patient care. The early orientation and specialization of aspiring doctors, beginning at the bachelor's level, must therefore be prioritized.^3^ Furthermore, the role of research is integral as it strengthens the academic foundation, improves oral and written communication skills and develops critical thinking capacity, particularly during the formative years of medical education.^5^ Exploring the concept of elective courses specifically tailored for medical students can shed light on its potential benefits.

## OUR EXPERIENCES

The board exam of second year MBBS had just finished when we came to know about the elective training organized by the Journal of Nepal Medical Association (JNMA), Kathmandu, through seniors and social media. Before enrolling to the month long elective training we had to fill the registration form which itself was interesting. Going through the interviews and finally being enrolled was a tremendous treat.

Joining a month of elective training held from 18 July 2022 to 18 August 2022, at Journal of Nepal Medical Association, NMA Building, Siddhi Sadan was eye-opening to second-year medical students from different parts of Nepal who were the program trainees. The daily session was for 7 hours, divided into three sections personality development, future career, and research and publication. The session adopted problem-based learning (PBL) strategy to sustain inclusiveness such that each participant had to work on effective communication skills, ability to teamwork, problem-solving skills, self-directed learning, ability to share information, acknowledge other points of view and identification of personal strengths and shortcomings. The skill of organizing an effective teaching-learning session was found to be developed among the students.

The trainees planned weekly adventure activities with careful pre-planning, task division, documentation, and execution to make each activity a learning opportunity with fun. Despite the pre planning, the trainees fell short in carrying out the task. Applying what they learned from the previous adventure activity helped them improve in the succeeding adventures. The same notion of documenting each activity in one's own career has assisted medical students in paving a road to go forward and adjust as needed in life.

The 4 am challenge was new and fascinating approach in which meeting place and time was decided the day before the event where every one of us had to be gathered. We travelled across the Kathmandu Valley's fringes during this challenge This activity had instilled in them the habit of getting up early and making the most of their morning time. Every member had the opportunity to lead at least one event, which helped them grow as a team, distribute tasks depending on individuals' strengths and weaknesses, and be impartial in their judgments. Despite the challenges, every adventure activity had learning for every one of us. Building resilience in these environments enabled them to endure through medical school hurdles with greater ease.

Emphasizing the importance of teamwork, we were mentored in collaborative projects to harness the collective talents, skills, and efforts of individuals. We felt that embracing teamwork can lead to greater success, productivity, and satisfaction in achieving shared goals. During a month, we got an opportunity to observe and be involved in programs like beyond medical school and JNMA hands on training for fast track publication. These programs focused on medical students, interns, and authors. Involving primarily in the managerial aspect and observing the academic part, we were engaged in one way or another. We got the opportunity to meet with interns and medical students of medical college outside the valley. This helped us to built a social network, and learn macro to micro-level management to run a program successfully.

## LEARNINGS

Working as a team and maintaining group dynamics has helped students to function in society, bringing unique knowledge, skills, and ideas. We grew from knowing nothing about research to learning about it and working on articles within one month of training. We got insight from seniors pursuing postgraduate careers in different countries.

### 1. Communication Skill

Communication is an important interpersonal skill that is mandatory for the doctor to develop good doctor-patient relationships. Listening to patients, providing information to the patient according to their understandable language and involving the patient in medical decisions is the prerequisite of a competent doctor and these qualities can be gained by enrolling in communication training.^4^ The daily session included presentations and discussions where each elective had an opportunity to put forward their point of view. Giving presentations and having discussions every day for a month helped to overcome stage fear and hesitancy. These sessions helped us to develop our communication skills. No matter how particular or general the engagement, effective communication and interpersonal interactions are prerequisites. These essential skills pertain to interprofessional cooperation, working as a team, and the interaction between patients and healthcare providers.

### 2. Leadership Skill

In this new age of teaching and learning, enrollment in skill labs has aided students in practical skills like leadership and communication.^4^ We learned the multidimensional aspects of leadership from the one-month elective training. Each of us was given opportunities to lead various events or google classroom activities. The training showed that leadership is about motivating the group and, developing temperament, and gaining knowledge. Leading the adventure activity and supervising every member involved, with proper work division, helped us to enhance leadership. Knowing the strengths and weaknesses of each member in the team helped us to assign work accordingly which led to better outcomes.

### 3. Teamwork and Social Networking

In order to minimize the disintegration of healthcare and its adverse effects on patients, clinicians must learn the art of cooperation from the very beginning of their training. While the World Health Organization stresses the value of doctors using a multidisciplinary team approach, medical students sometimes overlook collaborative work. As a result, teamwork needs to be incorporated into the undergraduate curriculum's guiding concepts.^6^ This training provided an open platform for medical students to work and learn from their colleagues in different medical colleges in the country. It cultivated a learning mindset, as team members learnt from one another's experiences and diverse perspectives.

### 4. Research

Research in the medical and health sciences advance medical knowledge, which leads to better health outcomes for patients. The session gave insight about 20 steps of research. The session of research training has encouraged us to start writing student articles and equip us with enough information required to conduct a research.

### 5. Career

Interaction with seniors pursuing postgraduate careers in the US, UK, India, Maldives, and Nepal has given us the idea of making lifechart and planning accordingly for career. The session on career enabled us to think about pull and push factors to be considered before deciding where to pursue our career.

## WAY FORWARD

Every medical student aspires to be a good doctor which requires knowledge, skills, hard work, and dedication. Medical students put such an effort to excel in their knowledge and skills that they forget the most crucial aspect of becoming a good doctor: seeing the patient as a human rather than the case we studied from a book. These kinds of courses help medical students, giving them an insight into knowledge beyond medical school, which is a prerequisite for every medical student. In this world of globalization, acquiring communication skills, leadership training, personality development and research involvement is essential for medical students. Students should be involved in different activities to help them be competent in their careers and social life. Strengthening soft skills and academics is pivotal for a comprehensive medical career.
